# Calcium Dynamics, WUSCHEL Expression and Callose Deposition during Somatic Embryogenesis in *Arabidopsis thaliana* Immature Zygotic Embryos

**DOI:** 10.3390/plants12051021

**Published:** 2023-02-23

**Authors:** Antonio Calabuig-Serna, Ricardo Mir, Jose M. Seguí-Simarro

**Affiliations:** Cell Biology Group—COMAV Institute, Universitat Politècnica de València, 46022 Valencia, Spain

**Keywords:** 2-deoxy-D-glucose, chlorpromazine, EGTA, FRET, in vitro culture, inositol 1,4,5-trisphosphate, ionophore A23187, W-7

## Abstract

In this work, we studied the induction of somatic embryogenesis in *Arabidopsis* using IZEs as explants. We characterized the process at the light and scanning electron microscope level and studied several specific aspects such as WUS expression, callose deposition, and principally Ca^2+^ dynamics during the first stages of the process of embryogenesis induction, by confocal FRET analysis with an *Arabidopsis* line expressing a *cameleon* calcium sensor. We also performed a pharmacological study with a series of chemicals know to alter calcium homeostasis (CaCl_2_, inositol 1,4,5-trisphosphate, ionophore A23187, EGTA), the calcium–calmodulin interaction (chlorpromazine, W-7), and callose deposition (2-deoxy-D-glucose). We showed that, after determination of the cotiledonary protrusions as embryogenic regions, a finger-like appendix may emerge from the shoot apical region and somatic embryos are produced from the WUS-expressing cells of the appendix tip. Ca^2+^ levels increase and callose is deposited in the cells of the regions where somatic embryos will be formed, thereby constituting early markers of the embryogenic regions. We also found that Ca^2+^ homeostasis in this system is strictly maintained and cannot be altered to modulate embryo production, as shown for other systems. Together, these results contribute to a better knowledge and understanding of the process of induction of somatic embryos in this system.

## 1. Introduction

Plant embryos are biological structures that aim to give rise to a new individual. Zygotic embryos are formed upon double fertilization and further zygote development, but this is not the only way to produce plant embryos. Embryos can also be produced artificially from immature male gametophytes, female gametes, or from vegetative (somatic) cells under certain in vitro conditions [1,2]. Somatic embryogenesis (the production of embryos from somatic cells) has been established as a model to study plant embryogenesis [1]. The first reports on somatic embryogenesis in *Daucus carota* date from 1958 [3,4]. More than 60 years since then, the number of available protocols for different species, from herbaceous crops to woody trees, has increased notably [5], and this process nowadays has a wide range of applications in plant biotechnology and breeding [6]. Therefore, the study of the mechanisms that regulate somatic embryogenesis will help give a better understanding of this process, and to generate improved protocols to further exploit its benefits.

Somatic embryos are produced either directly from the explant or indirectly through an intermediate callus phase. In *Arabidopsis thaliana,* somatic embryogenesis can be in vitro induced from protoplasts [7], root explants [8], shoot apical tip and floral bud explants [9], shoot apex explants of young seedlings [10], or germinating embryos from mature seeds [11]. However, the best-known and studied system of somatic embryogenesis in *Arabidopsis* uses immature zygotic embryos (IZEs) at the late cotyledonary stage as explants [12]. It was described that, when in vitro cultured on auxin-containing medium, IZEs directly develop somatic embryos on the adaxial proximal ends of cotyledons within two weeks of culture [13,14]. Longer culture time results in the formation, on the cotyledon abaxial side, and of a callus-like structure that generates somatic embryos through indirect embryogenesis [13]. All other embryo regions were described as non-embryogenic [13,15]. Histological analysis showed that somatic embryos arise from protodermal and subprotodermal cell layers [13]. The establishment of totipotency in these cells and their reprogramming towards embryogenesis was reported to be mediated by a decrease of auxin signaling and their symplasmic isolation from the non-embryogenic neighboring cells by callose deposition at plasmodesmata [15]. At the genetic level, somatic cell dedifferentiation and activation of the embryogenic pathway is a complex process that implies alterations of the transcriptional activity, turning off the expression of some specific genes and activating several others, principally embryo identity genes [3,16]. Specific master regulators involved in the activation of somatic embryogenesis have been identified [17,18]. Specifically, overexpression of *WUSCHEL (WUS)*, a homeodomain protein characterized by its role in the shoot apical meristem maintenance, promotes the occurrence of somatic embryogenesis [8,19]. The expression of *WUS* [19], and of several other embryo marker genes such as *BABYBOOM* [20], *LEC2* [13], *SERK* [21], and *WOX2* (a *WUS*-related homeobox gene), has been used to determine the spatio-temporal patterns of this process.

Although the last decade has witnessed significant advances in the elucidation of the genetic networks and the epigenetic mechanisms operating for the formation of somatic embryos [3,17], the intracellular signal that triggers somatic embryogenesis is not clearly determined. Ca^2+^ is one of the most important secondary messengers in plant cell signal transduction processes, controlling gene expression upon binding to calmodulin (CaM) or other Ca^2+^-sensing proteins [22], and further interaction with transcription factors. The concentration of free resting cytosolic Ca^2+^ in plant cells is usually kept very low, within the range of 50–100 nM, being higher in the different cell compartments considered as Ca^2+^ reservoirs, namely the vacuole, the ER, and the apoplast [23,24]. Due to its cellular toxicity even at low concentrations, the role of Ca^2+^ as a secondary messenger is based on the generation of concentration gradients and transient increases of cytosolic concentrations [25]. Thus, a possible scenario for induction of somatic embryogenesis could be cytosolic auxin-mediated Ca^2+^ that increases acting as a rapid activator of embryo identity genes. Indeed, a number of studies in different species, such as *Musa* [26], *Cocos nucifera* [27], *Hevea brasiliensis* [28], and *Daucus carota* [29], among others, support the notion that direct Ca^2+^ supplementation of in vitro media enhances somatic embryogenesis, therefore pointing to an important role of this secondary signal as inducer of somatic embryogenesis. Other studies, however, suggest that increased Ca^2+^ levels are not positive to increase production of somatic embryos [30,31,32,33]. For this reason, the study of Ca^2+^ dynamics during the first stages of this morphogenic process would give us valuable information about the factors governing the induction of somatic embryogenesis and, in particular, to decipher the role of Ca^2+^ in this process.

Due to the universal role of Ca^2+^ as a molecular signal, some tools have been developed to monitor Ca^2+^ levels in a broad range of biological systems. In *Arabidopsis*, the study of Ca^2+^ dynamics can be greatly facilitated by the use of *cameleon* lines. *Cameleon* constructs are based on the principle of fluorescence resonance energy transfer (FRET) that occurs between two fluorophores when they become spatially closer. This happens when the linker protein, usually CaM in the case of *cameleons*, binds to Ca^2+^ and undergoes a conformational change that approaches the donor fluorophore to the acceptor. Hence, when the donor is excited, it transfers a certain amount of energy to the acceptor, which becomes excited itself, emitting fluorescence. Krebs et al. [34] developed a collection of *Arabidopsis* lines that express the *cameleon* construct specifically in certain cell regions, such as the cytoplasm or the plasma membrane, using cyan fluorescent protein (CFP) as a donor fluorophore and yellow fluorescent protein (YFP) as an acceptor. They are therefore excellent Ca^2+^ sensors. However, these lines have not yet been used to study Ca^2+^ dynamics during somatic embryogenesis.

Despite the importance of *Arabidopsis* as a model to study somatic embryogenesis [1] and the role of Ca^2+^ in this process, it is surprising that, to the best of our knowledge, no studies of Ca^2+^ distribution or chemical modulation during somatic embryogenesis have been published in *Arabidopsis*. In this work, we studied Ca^2+^ dynamics during somatic embryogenesis from *Arabidopsis* IZEs. Using wild type, *WUS-reporter* and *cameleon*-transformed lines, we performed a multidisciplinary study including a characterization of the different stages through scanning electron microscopy (SEM), confocal microscopy for *WUS-reporter* expression and FRET-based Ca^2+^ imaging in *cameleon* lines, and a pharmacological study where different chemicals known to interfere with Ca^2+^ homeostasis and signaling were applied to embryogenic cultures. Our results shed light on the different origins of the IZE-derived somatic embryos, the embryogenic nature of the newly proliferating tissues, the distribution of Ca^2+^ during the initial stages of embryogenesis induction, and the role of Ca^2+^ and CaM modulation in this process.

## 2. Results

### 2.1. Induction of Somatic Embryogenesis from IZEs of Arabidopsis Cameleon Lines

**Figure 1:**
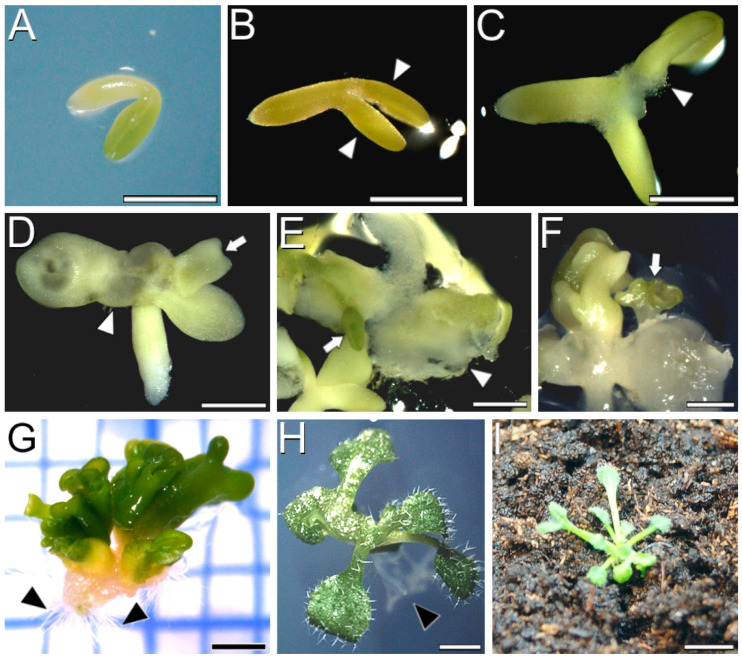
Induction of somatic embryogenesis from YC3.6-Bar *cameleon Arabidopsis* IZEs. (**A**) Freshly isolated immature cotyledonary embryo. (**B**) Four-day-old cultured IZE with thickened cotyledons (white arrowheads). (**C**) Formation of protrusions (white arrowhead) in the adaxial side of cotyledons. (**D**) Formation of a protrusion (white arrowhead) in the adaxial side of the **left** cotyledon, and early emergence of a somatic embryo from the adaxial side of the **right** cotyledon (arrow). (**E**) Development of a callus mass from a cotyledonary protrusion (white arrowhead) and of a direct somatic embryo (arrow). (**F**) Somatic embryos (arrow) developed from a callus mass derived from a protrusion after 14 days of culture. (**G**) Cluster of somatic embryos excised from the explant after 21 days of culture. Black arrowheads point to the radicles formed at the basal pole of the cluster. (**H**) Germinated in vitro plantlet from an individualized somatic embryo. Black arrowhead points to roots. (**I**) *Arabidopsis* plant transferred to soil and acclimatized. Bars: (**A**–**F**) 500 µm; (**G**,**H**) 1 mm; (**I**) 5 mm.

We used *Arabidopsis* seeds transformed with the *cameleon* YC3.6-Bar construct to isolate and in vitro culture IZEs at the cotyledonary stage (Figure 1A). First, we checked the embryogenic response of the *cameleon* lines, comparing it with Col-0 wild type plants. The embryogenic response of the two lines was not statistically different (Supplementary Figure S1), thus confirming the validity of these *cameleon* lines to study somatic embryogenesis under our experimental conditions. Four days after isolation, IZEs enlarged in general, but principally in the proximal region of the cotyledons, which induced their separation (Figure 1B). Following that, visible embryogenic protrusions developed from the cotyledon nodes at the proximal adaxial region (Figure 1C). Occasionally, embryogenesis was directly induced from the surface of the cotyledon (arrows in Figure 1D,E). However, the most frequent scenario was the growth of embryogenic protrusions in the form of a cell mass (Figure 1E, arrowhead). From these cell masses, clusters of embryos were formed after 14 days of culture (Figure 1F, arrow). Upon excision of these clusters from the explant, by day 21 of culture they turned an intense green, continued elongating, and developed radicles (Figure 1G). Their cotyledons, however, did not develop as much as those of the IZEs used as explants. Individualization of somatic embryos allowed for their germination into a rooted, regenerated plantlet (Figure 1H) which, upon transference to soil and acclimatization, became a complete *Arabidopsis* plant in 15 days (Figure 1I).

### 2.2. Scanning Electron Microscopy of Somatic Embryogenesis from Arabidopsis IZEs

**Figure 2:**
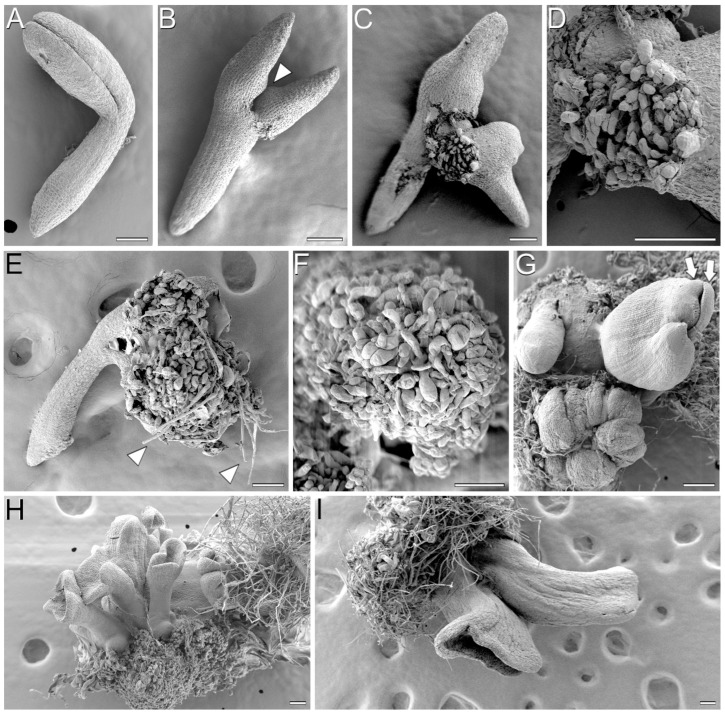
SEM analysis of somatic embryogenesis from YC3.6-Bar *cameleon Arabidopsis* IZEs. (**A**) Isolated immature cotyledonary embryo after three days of culture. (**B**) Five-day-old cultured IZE with embryogenic protrusions (white arrowhead) in the adaxial proximal region of cotyledons. (**C**,**D**) Mass of proliferating cells emerged from the protrusion upon burst of the cotyledon epidermis at day 7 of culture. (**E**,**F**) Growth of the cell masses from both protrusions into a single mass that covers both cotyledons. Note the occurrence of the first radicles (arrows). (**G**) Development of somatic embryos at different developmental stages from the surface of the callus mass. Arrows point to the two cotyledons, still closed, of a bent torpedo embryo. (**H**) Cluster of somatic embryos excised from the explant after 14 days of culture. (**I**) Detail of two trumpet-shaped, elongated cotyledonary embryos. Bars: 100 µm.

In order to have a closer view of the process of embryogenesis induction, we processed samples of IZEs at different stages during SE for analysis with FESEM. After three days of in vitro culture (Figure 2A), no remarkable changes with respect to in vivo IZEs were observed. After 5 days, growth from inner cells of the apical region of the hypocotyl and principally the adaxial proximal region of the cotyledons was evident, and small protrusions arose (Figure 2B, arrowhead), in some cases producing ruptures of the protodermis. Later (around day 7), the massive growth of the protrusions forced the rupture of the covering protodermal layer (Figure 2C) and the emergence of a mass of proliferating cells (Figure 2D). Further growth of the cell masses from both cotyledons may fuse into a single mass that frequently covers both cotyledons (Figure 2E,F). In these cell masses, differentiation of some organs such as root-like structures could be distinguished (Figure 2E arrowheads). Some of these structures continued their growth on the surface of the cell mass, becoming differentiated embryos (Figure 2G). Elongated, cotyledonary-like embryos were clearly visible emerging on the surface of the cell masses after 14 days of culture (Figure 2H). These embryos, however, frequently showed short cotyledons closely apposed or even fused by their margins, forming trumpet-shaped structures (Figure 2I).

**Figure 3:**
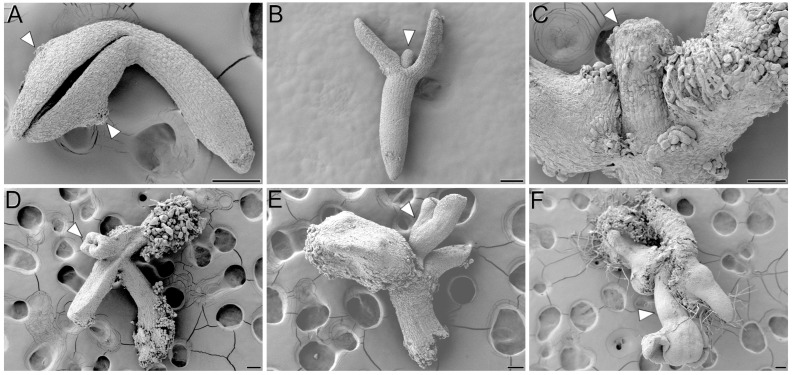
SEM analysis of somatic embryogenesis from YC3.6-Bar *cameleon Arabidopsis* IZEs. (**A**) Cultured IZE after 3 days of culture. Note the occurrence of protrusions at the abaxial sides of both cotyledons (arrowheads). (**B**) Cultured IZE with a finger-like protrusion at the shoot apical meristem (arrowhead). (**C**) 14-day-old IZE with an elongating finger-like protrusion at the shoot apical meristem (arrowhead). (**D**–**F**) 14-day-old IZEs developing a secondary somatic embryo (arrowhead) from the shoot apical meristem. Bars: 100 µm.

In addition to the formation of protrusions at the adaxial proximal regions of cotyledons, we also observed the occasional formation of protrusions at the cotyledon abaxial regions (Figure 3A) and, more frequently, at the shoot apical meristem region, where a finger-like appendix emerged (Figure 3B) after activation of growth at the cotyledon adaxial regions. In some IZEs, these protrusions elongated (Figure 3C) and, after some days of culture, transformed into secondary somatic embryos (Figure 3D–F) similar to those formed from the cotyledon adaxial regions, occurring at a lower frequency.

### 2.3. Expression of the WUS-Reporter upon Induction of Somatic Embryogenesis in Arabidopsis

**Figure 4:**
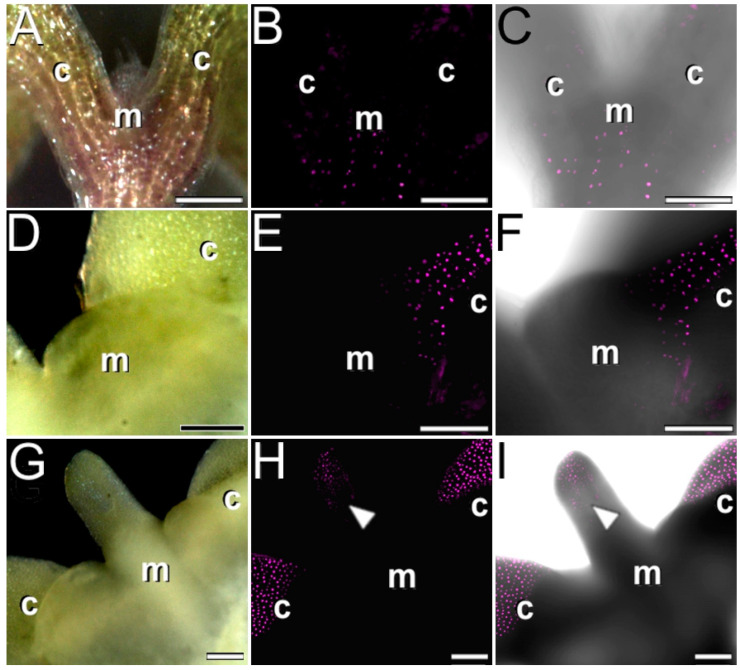
Induction of somatic embryogenesis in IZEs of an *Arabidopsis* line expressing a *WUS*-reporter. Each row of images shows the same field imaged in the binocular microscope by bright field (**left**), fluorescence (**center**), and merge of both signals (**right**). (**A**–**C**) Control IZE cultured in non-embryogenic conditions. Note that the *WUS* signal is limited to the central zone of the shoot apical meristem (m). (**D**–**F**) 5-day-old IZE cultured in somatic embryogenesis medium showing WUS expression only at the swollen adaxial proximal region of the cotyledon (c). (**G**–**I**) 7-day-old IZE showing *WUS* expression at the enlarged appendix (arrowhead) of the shoot apical meristem (m) and the protrusions emerged from the adaxial proximal regions of both cotyledons (c). Bars: 100 µm.

To evaluate the embryogenic nature of the growth and proliferation observed in the cotyledon adaxial proximal region and the shoot apical meristem, we induced somatic embryogenesis from IZEs of an *Arabidopsis WUS*-reporter line. As a control, we also cultured IZEs in hormone-free medium, which was unable to induce somatic embryogenesis. In five-day-old control IZEs, *WUS*-reporter expression was confined to the central zone of the shoot apical meristem, as expected (Figure 4A–C). In IZEs cultured in inductive medium (with 2,4-D), *WUS*-reporter expression at day 5 was observed only in cells of the cotyledon nodes, immediately after their initial swelling to form protrusions (Figure 4D–F), confirming the induction of embryogenesis in these cells. At this stage we also observed a parallel silencing of *WUS*-reporter expression in the central cells of the shoot apical meristem. In IZEs at day 7, a finger-like appendix emerged from the shoot apical meristem region (Figure 4G), *WUS*-reporter expression was also found in cells of the tip of the finger-like appendix (arrowhead in Figure 4H,I), in addition to the mesophyll cells of the protrusions at the cotyledon nodes. The rest of the explant did not show any detectable *WUS*-reporter expression. Together, these results confirm that, upon induction of somatic embryogenesis, the protodermal and subprotodermal cells of the cotyledon nodes are first reprogrammed to embryogenesis. Later on, and when present, cells from the finger-like appendix of the shoot apical meristem are also reprogrammed to embryogenesis.

### 2.4. Callose Staining during Somatic Embryogenesis in Arabidopsis

**Figure 5:**
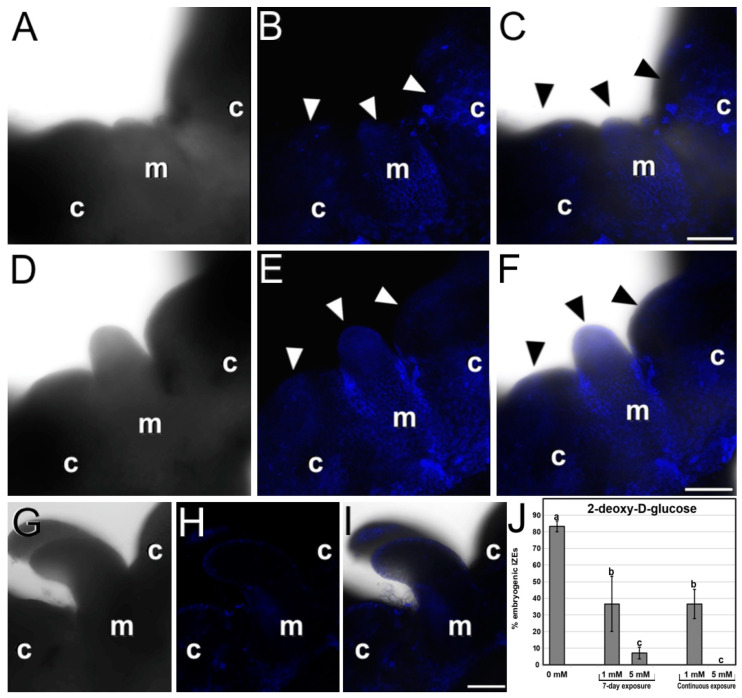
Callose staining with aniline blue cultured IZEs. Each row of images shows the same field of aniline blue-stained samples imaged by bright field (**left**), fluorescence (**center**), and merge of both signals (**right**). (**A**–**C**) Five-day-old IZE with aniline blue staining (arrowheads) in the cotyledon nodes (c) and in the shoot apical meristem region (m). (**D**–**F**) IZE with aniline blue staining (arrowheads) in the cotyledon nodes (c) and in the shoot apical meristem region (m) where the finger-like appendix is also stained. (**G**–**I**) IZE with a somatic embryo growing from the finger-like appendix with almost no aniline blue staining in the cotyledon nodes (c) nor in the shoot apical meristem region (m). (**J**) Effect of 2-deoxy-D-glucose (applied during the first 7 days and continuously) in the percentage of embryogenic IZEs produced (% embryogenic IZEs) out of the total of embryos cultured. Different letters represent significant differences according to the LSD test. Bars: 100 µm.

We used aniline blue to stain cultured IZEs and study callose distribution during the induction of somatic embryogenesis. It was described that during the second week of culture (around day 10), callose was synthesized in the cell walls of cells of the cotyledon protrusions as they grow and switch towards embryogenesis [15]. In our IZE cultures, we also observed a similar increase in callose deposition at the cells of cotyledonary protrusions, as revealed by aniline blue staining. However, in our samples, such an increase was observed around day 5 of culture (Figure 5A–C), indicating that, at least in our IZE culture system, somatic embryogenesis seems to proceed faster. In addition, in some IZEs we also observed a clear aniline blue staining at the shoot apical meristem region, before the appearance of the finger-like appendix, and during the first stages of appendix elongation (Figure 5D–F). However, in growing embryos emerged for the appendix, aniline blue staining was notably reduced (Figure 5G–I). Together, these results confirm that callose accumulates in the cells undergoing the embryogenic switch in the two cotyledonary protrusions, and in the shoot apical meristem region as it transforms into a finger-like embryogenic appendix. We also evaluated the role of callose during the process by inhibiting callose synthesis with 2-deoxy-D-glucose (Figure 5J) and found that, for all concentrations and durations tested, inhibition of callose synthesis was seriously detrimental for embryo production, being almost null for 5 mM. The percentage of embryogenic IZEs was similar for 7-day and continuous 2-deoxy-D-glucose exposures, indicating that the role of callose on embryogenesis induction is exerted during the first week of culture, having no relevant effect after the first week. Together, these results show that callose deposition during the first week of culture is essential for a successful induction of somatic embryogenesis, being abundantly deposited at embryogenic regions prior to the development of somatic embryos.

### 2.5. FRET Imaging of Ca^2+^ Distribution during Somatic Embryogenesis in Arabidopsis

**Figure 6:**
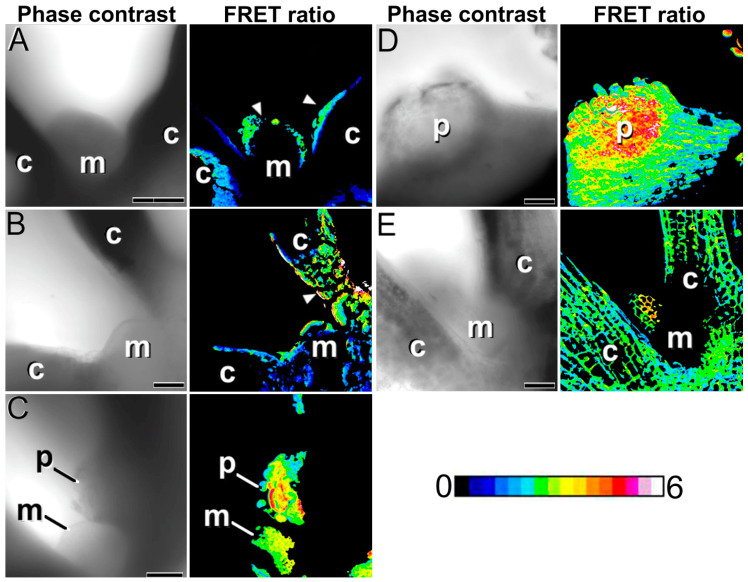
FRET imaging of Ca^2+^ signaling during the induction of somatic embryogenesis in YC3.6-Bar *cameleon Arabidopsis* IZEs. Each pair of images show the same field imaged by phase contrast (**left**) and by the FRET (YFP/CFP emissions) ratios. The LUT bar displays the false coloration of FRET ratios. (**A**) Shoot apical meristem (m) and proximal region of the cotyledons (c), showing increased Ca^2+^ levels in the outermost cell layer of the shoot apical meristem and in the epidermis of the adaxial proximal cotyledon region (arrowheads). (**B**) Cells of the mesophyll region (arrowhead) of the cotyledon (c). (**C**) Shoot apical meristem (m) and a protrusion (p) at the adaxial proximal cotyledon region showing high Ca^2+^ levels. (**D**) Large protrusion (p) at the adaxial proximal region of the cotyledon with a radial gradient of Ca^2+^ levels, being higher at the center of the protrusion, where somatic embryos are being formed. (**E**) Shoot apical meristem (m) and proximal region of the cotyledons (c) of an IZE cultured in non-embryogenic conditions. Note the homogeneous distribution of Ca^2+^ in the regions imaged. Bars: 60 µm.

We performed a FRET study to track the dynamics of Ca^2+^ during the process of induction of somatic embryogenesis in our *Arabidopsis cameleon* lines (Figure 6 and Supplementary Figure S2). The first sign of change in the Ca^2+^ levels after the onset of in vitro culture was an increase in the protodermal cell layer of the shoot apical meristem region and the cotyledon nodes (arrowheads in Figure 6A), outlining the regions where embryogenic cell proliferation will take place. Then, the Ca^2+^ signal persisted in the protoderm and increased in the inner cells of the cotyledon node region (Figure 6B), while the rest of the IZE did not show relevant changes in Ca^2+^ signal. Once the protrusions were evident at the surface of the adaxial proximal cotyledon region, the Ca^2+^ signal markedly increased in these regions, as well as in the enlarged shoot apical meristem appendix (Figure 6C). Large protrusions (Figure 6D) and appendices showed the highest levels of Ca^2+^, which were much higher than in any other IZE region. Ca^2+^ signal in the protrusions was distributed in a gradient manner (Figure 6D), with less signal at the periphery of the protrusion and more intense signal at the center of the protrusion, which is the place where new embryogenic structures are being formed (Figure 2D). In parallel, we cultured IZEs in hormone-free medium, unable to induce somatic embryogenesis, and observed their Ca^2+^ signal in the cotyledons and shoot apical meristem. As seen in Figure 6E, Ca^2+^ signal of non-induced IZEs was remarkably homogeneous, having cotyledons and shoot apical meristem levels of Ca^2+^ similar to those of the rest of the IZE. Together, these results show that Ca^2+^ levels increase in the cotyledon node and the shoot apical meristem, prior to the occurrence of somatic embryogenesis, with a pattern remarkably similar to that of *WUS*-reporter expression, making Ca^2+^ increase an early marker of somatic embryogenesis. Later on, Ca^2+^ levels increase even more in the protrusions in general and particularly in embryogenic cells, showing that high Ca^2+^ levels are necessary for an efficient transition of somatic cells into somatic embryos.

### 2.6. Modulation of Intracellular Ca^2+^ Levels

**Figure 7:**
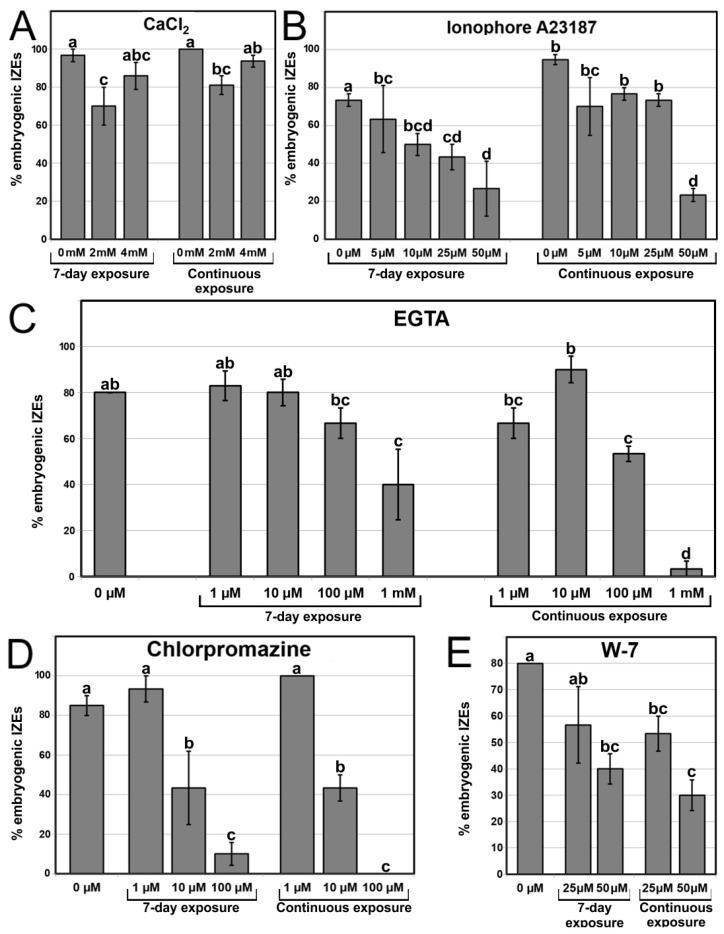
Modulation of intracellular Ca^2+^ levels with different chemicals: CaCl_2_ (**A**), ionophore A23187 (**B**), EGTA (**C**), chlorpromazine (**D**), and W-7 (**E**). Chemicals were used at different concentrations and for 7-day and continuous exposures. The effect of each treatment is expressed as the percentage of embryogenic IZEs produced (% embryogenic IZEs) out of the total of embryos cultured. Different letters represent significant differences according to the LSD test.

**Figure 8:**
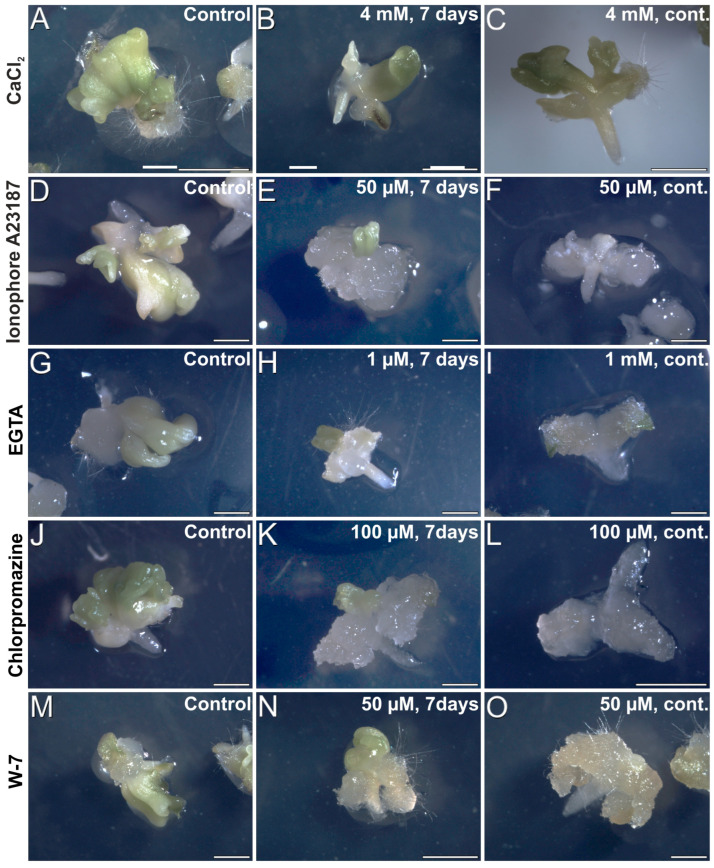
Carrot somatic embryos produced in cultures with different added chemicals, including CaCl2 (**A**–**C**), ionophore A23187 (**D**–**F**), EGTA (**G**–**I**), chlorpromazine (**J**–**L**), and W-7 (**M**–**O**) at different concentrations and durations, as described in the images. Bars: 1 mm.

We performed a pharmacological study to modulate the intracellular Ca^2+^ levels. We treated embryogenic cultures with different chemicals known to interfere with intracellular Ca^2+^ levels, observed the embryos produced, and calculated the percentage of embryogenic IZEs for each treatment. First, we applied compounds known to increase Ca^2+^ levels in other somatic embryogenesis systems. We added different CaCl_2_ concentrations (2 and 4 mM) and compared the embryo production with that of control cultures with 1.02 mM CaCl_2_, the standard CaCl_2_ concentration of the induction medium (Figure 7A). CaCl_2_ addition did not show any positive effect in terms of percentage of embryogenic IZEs produced, not during a 7-day exposure or during continuous exposure. Even with the highest concentration, the morphology of both the IZEs and the somatic embryos produced was similar to controls (Figure 8A–C). The use of ionophore A23187, a plasma membrane Ca^2+^ channel used to alter intracellular Ca^2+^ gradients [35], produced no positive effects at any concentration or duration, but produced negative effects at higher concentrations in terms of reduced percentages of embryogenic IZEs (Figure 7B) and of proliferation in IZEs of callus masses instead of somatic embryos (Figure 8D–F). In line with this, the addition of InsP_3_, known to induce Ca^2+^ efflux from different intracellular stores such as the ER or vacuoles [36], did not produce any significant change, positive or negative, in the percentage and morphology of embryogenic IZEs, or at any of the concentrations used (0.1, 1, 10 and 100 µM). In summary, none of these compounds were effective for increasing embryogenesis. Instead, the effect was negative in some cases.

To reduce intracellular Ca^2+^ levels we used EGTA, a highly specific Ca^2+^ chelator, which at 1 mM caused a significant inhibition of embryogenesis for both exposure times, inhibiting about 50% when applied for 7 days, and almost completely when applied continuously (Figure 7C). IZE morphology was also severely affected, being almost totally covered by callus tissue (Figure 8G–I). We also used chlorpromazine and W-7, two CaM antagonists, to interfere with Ca^2+^-CaM signaling. The inhibition of CaM with chlorpromazine (Figure 7D) and W-7 (Figure 7E) caused a dose-dependent inhibition of embryogenesis for both durations, which was almost complete when chlorpromazine was applied at 100 µM continuously. Consistent with this, the morphology of IZEs was dramatically altered, with almost no signs of somatic embryo growth and the development of callus masses with both chlorpromazine (Figure 8J–L) and W-7 (Figure 8M–O). In summary, both reducing the levels of intracellular Ca^2+^ or interfering with Ca^2+^ binding to CaM, negatively affects the induction and growth of somatic embryos, producing very similar patterns of callus growth and reduction of the percentages of embryogenic IZEs.

## 3. Discussion

### 3.1. The Shoot Apical Meristem of IZEs also Produces Somatic Embryos

Our microscopical analysis of somatic embryogenesis from IZEs showed the direct development of embryos from the cotyledon surface together with the development of inner cell masses that give rise to embryo clusters (Figure 1 and Figure 2), which is consistent with their reported protodermal and subprotodermal origin [13], respectively. We also observed the occasional development of protrusions in the abaxial side of the cotyledons (Figure 3A), which may possibly come from the same inner cells of the cotyledon node that, for any reason (difficult cotyledon separation, for example), cannot emerge from the adaxial side. This is also in line with the reported formation of a callus-like structure on the cotyledon abaxial side that may indirectly produce somatic embryos [13]. However, we also consistently observed that, once established the cotyledon protrusions, some IZEs developed, at the shoot apical meristem region, an appendix that eventually produced somatic embryos (Figure 3). This is surprising because it was never reported in the previous literature describing this system. Pioneering studies [12] did not mention anything about the involvement of the IZE shoot apical meristem in somatic embryogenesis. However, it is interesting to note that in one of their images, different cultured IZEs with finger-like appendices are clearly observed. In other, more recent works, the shoot apical meristem is described as not being involved in somatic embryo formation [13,15]. However, it is also interesting to note that in their analysis of IZEs expressing *WOX2:YFP,* an enlarged structure at the shoot apical meristem region showed an intense *WOX2* signal, consistent with the *WUS* expression we hereby show. Thus, our light microscopy and SEM analyses, together with our data on *WUS* expression and calcium dynamics during the establishment of embryo identity in IZE cells, clearly demonstrate that the appendix formed at the shoot apical meristem is also capable of producing somatic embryos. This is not surprising, since induction of somatic embryogenesis was demonstrated to be possible from shoot apex explants excised from 4–5-day-old seedlings [9,10].

### 3.2. High Ca^2+^ Levels Act as a Trigger of Somatic Embryogenesis, Marking the Onset of the Process

During sexual plant reproduction, zygotic embryogenesis is initiated with two defined Ca^2+^ increases, the so-called Ca^2+^ signature of initiation of embryogenesis [37]. First, there is a short cytoplasmic Ca^2+^ transient increase (oscillation) in the egg and central cells, associated with pollen tube burst and the discharge of sperm cells. Then, there is a second, prolonged Ca^2+^ increase exclusive for the egg cell, associated with successful egg fertilization [37]. It is assumed that the developmental programs of zygotic and somatic embryogenesis are very similar, if not indistinguishable [38]. Thus, one can expect that in the somatic cell to be reprogrammed to embryogenesis, a similar Ca^2+^ signature should also be observed as a trigger for embryogenesis initiation. We were not able to identify the first, short Ca^2+^ transient peak at the onset of somatic embryogenesis, most likely due to its short duration, and principally because it is associated to pollen tube discharge [37], which does not apply in this context. However, we observed a prolonged Ca^2+^ increase in the embryogenic regions that initiated in the protoderm and extended to the inner cells of the cotyledon nodes and the shoot apical meristem appendix (Figure 6), coinciding with regions with cells expressing the *WUS*-reporter (Figure 4). Thus, Ca^2+^ increase is an early marker of the onset of somatic embryogenesis. Interference with Ca^2+^ signaling by inhibiting CaM with two CaM antagonists, W-7 and chlorpromazine, led to a significant decrease of the percentage of embryogenic IZEs and a dramatic alteration of their morphology (Figure 7D,E and Figure 8J–O). The fact that 7-day and continuous treatments with CaM antagonists resulted in a similar reduction of embryo production may indicate that the signaling role of Ca^2+^ is principally exerted during the inductive stage of the embryogenic process, which strengthens the notion of a critical role of Ca^2+^ as a triggering element for somatic embryogenesis. We therefore postulate that this would be the Ca^2+^ signature in somatic embryogenesis equivalent to the zygotic counterpart.

### 3.3. Ca^2+^ Homeostasis Cannot Be Altered to Induce Somatic Embryogenesis from IZEs

The role for Ca^2+^ in the induction of zygotic embryogenesis has been widely acknowledged [37]. In somatic embryogenesis, several reports have documented the need for defined, constant Ca^2+^ levels for this process to occur in different species [31,32,33]. Other reports, however, have documented that alteration of the intracellular Ca^2+^ levels has direct consequences in the rate of embryogenesis induction. In some of these cases, increasing Ca^2+^ levels had a positive impact in embryo production and reducing them was detrimental for embryo production [26,27,28,29], whereas in others, a reduction of Ca^2+^ levels was beneficial for somatic embryogenesis [39]. In this work, we used different pharmacological approaches to modulate intracellular Ca^2+^ levels. With the addition of CaCl_2_ to the medium, no positive results were observed in any case (Figure 7A and Figure 8A–C), which suggests that Ca^2+^ influx is tightly regulated at the plasma membrane level, since increasing the intracellular–extracellular Ca^2+^ gradient had no effect. In turn, alteration of intracellular Ca^2+^ levels with ionophore A23187 produced dramatic, dose-dependent negative effects in IZE morphology in their competence to produce somatic embryos (Figure 7B and Figure 8D–F). On the other hand, reducing the levels of available Ca^2+^ by EGTA chelation produced similarly negative and dose-dependent results (Figure 7C and Figure 8G–I).

Together, these results show that influx or efflux of even small amounts of Ca^2+^ have important consequences in the maintenance of Ca^2+^ homeostasis, as expected considering the typically very low cytosolic Ca^2+^ concentrations (50–100 nM; [23]). For the particular case of induction of somatic embryogenesis from *Arabidopsis* IZEs, a strict control of Ca^2+^ homeostasis is required, and the efficiency of the process cannot be improved by increasing intracellular Ca^2+^ levels, as occurs in other, more plastic systems described above. It seems that modulation of somatic embryogenesis by altering Ca^2+^ levels is not a common feature for all somatic embryogenesis systems. It is possible in some systems, like *Musa* [26], *Cocos nucifera* [27], *Hevea brasiliensis* [28], or *Daucus carota* [29], but not in others like *Santalum album* [31], *Pinus patula* [32], *Coffea canephora* [33], and *Arabidopsis*, as we hereby show. However, the reason why different species behave differently remains to be elucidated.

### 3.4. Somatic Cells Transition to Embryogenesis First at the Cotyledon Protrusions and then at the Tip of the Shoot Apical Meristem Appendix

*WUS* is a transcription factor defined as a master regulator in plant growth signaling due to its key role in the regulation of both embryogenic and meristematic stem cells [18]. In *Arabidopsis* plants and zygotic embryos, *WUS* is typically expressed in the central zone of the shoot apical meristem [40]. Upon induction of somatic embryogenesis, *WUS* is also expressed in the newly induced embryogenic cells even before they transform into embryos [18,19], which makes *WUS* an early marker of the developmental transition from vegetative to embryogenic development. We used *Arabidopsis WUS* lines to check for the induction of primary somatic embryos from IZEs. Early *WUS* expression in the protrusion-producing protodermal and subprotodermal cells of the cotyledon node (Figure 4D–F) was accompanied by deposition of callose (Figure 5A–C) and increased calcium levels (Figure 6B) in these cells, which confirms the embryogenic nature of the cells of these protrusions and the involvement of calcium and callose at the onset of this process. Previously, callose deposition was shown as essential to isolate the embryogenic domains from the rest of the explant [15], and Ca^2+^ increases could be related with both triggering of embryogenesis and the activation of callose synthesis by Ca^2+^-dependent callose synthases, as reported for other in vitro embryogenesis systems [41]. However, deposition of callose and increased calcium levels were not paralleled by *WUS* expression at the shoot apical meristem region, which is consistent with the absence of somatic embryos directly produced from cells of the shoot apical meristem. This suggests that the role of Ca^2+^ and callose in this region would not be related with triggering of somatic embryogenesis. Instead, it could be speculated that Ca^2+^-mediated callose deposition at the shoot apical meristem would be needed to isolate these cells not for the establishment of embryo identity, but for the elimination of the stem cell identity of shoot meristem cells in order to allow for their growth. Indeed, successful induction of somatic embryogenesis in IZEs from different *Arabidopsis* mutants lacking embryonic shoot apical meristems demonstrated that a functional shoot meristem is not necessary for the induction of somatic embryos [38]. At later culture stages, when cotyledonary protrusions are clearly visible and a large, finger-like appendix emerges at the shoot apical meristem region, cells of the tip of this appendix begin to express *WUS,* as revealed by the expression of the *WUS*-reporter (Figure 4G–I) with a pattern similar to that described for *WOX2* [15], and somatic embryos are produced from these cells. Thus, the transition to embryogenesis in IZEs would take place in two steps: first in protodermal and subprotodermal cells of the cotyledon nodes, which is accompanied by a loss of stem cell identity in the shoot apical meristem, and then in the tip of the finger-like appendix developed from the shoot apical meristem.

### 3.5. Concluding Remarks

In this work, we studied the process of induction of somatic embryogenesis from *Arabidopsis* IZEs. Some of the results presented here, including the changes undergone by the cotyledonary nodes to become embryogenic regions, are in line with those previously shown by other authors [12,13,14,15]. However, there are some others that, to the best of our knowledge, have not been reported before and may be relevant for a better knowledge and understanding of the system. First, we observed that, at least in our hands, this system develops faster than reported in other cases, as the events associated with callose deposition previously described around day 10 [15] were observed in our cultures around day 5. This was confirmed by our light microscopy and SEM observations, and by the 2-deoxy-D-glucose experiments (Figure 5J), which confirmed that callose deposition during the first week of culture is essential for successful embryo induction. We also showed that, in addition to the transformation of the cotyledon nodes into embryogenic regions where somatic embryos emerged from, a finger-like appendix develops from the shoot apical meristem region, and somatic embryos are, to a lesser extent, also generated from this appendix. We demonstrated that, as opposed to other somatic embryogenesis systems, Ca^2+^ homeostasis in *Arabidopsis* IZEs is strictly maintained and cannot be altered. Finally, we showed the dynamics of Ca^2+^ in the embryogenic regions, assigning putative roles in the activation of callose deposition and the induction of somatic embryogenesis. Together, these results contribute to a better understanding of this fascinating morphogenic process.

## 4. Materials and Methods

### 4.1. Plant Materials

We used *Arabidopsis thaliana* (Col-0) wild type and transgenic lines for expressing the YC3.6-Bar *cameleon* construct [34] carrying a signal for cytoplasm targeting, kindly provided by Prof. Jörg Kudla (Münster University, Münster, Germany). To analyze the spatial distribution of *WUS* expression, we used the *A. thaliana* transgenic line pCLV3:GFP-ER_pWUS:DsRED-N7 (NASC ID: N23895), kindly provided by Dr. Cristina Ferrándiz (IBMCP-CSIC, Valencia, Spain). This *WUS*-reporter line expresses the DsRED fluorescent protein under the control of the *WUS* promoter.

### 4.2. Induction of Somatic Embryogenesis

Eight week-old silique-producing *Arabidopsis* plants were used as donors of explants. Siliques were harvested and surface-sterilized for 30 s in 70% ethanol and 20 min in 10% commercial bleach, followed by three rinses in sterile distilled water. Under a binocular microscope, siliques were dissected to isolate the immature seeds. IZEs with fully developed, bent, and green cotyledons were used as explants. They were rescued by carefully removing the seed coat and the endosperm, and in vitro cultured as previously described [14]. IZEs were transferred to culture dishes with induction medium (Table 1). Dishes were kept at a 25 °C, 16/8 photoperiod for 15 days. Then, the induced somatic embryos were excised from the explant and transferred to germination medium (Table 1). Germinated plantlets were transferred to soil and acclimated at 25 °C in a 16/8 photoperiod.

### 4.3. Scanning Electron Microscopy

For scanning electron microscopy, we processed samples of *Arabidopsis* IZEs cultured in vitro during 3, 5, 7, and 14 days in solid E5 medium. Samples were fixed in Karnovsky fixative as previously described [44] for 5 h at room temperature under vacuum conditions, rinsed three times (30 min each) in 0.025 M cacodylate buffer, and kept in 0.025 M cacodylate buffer at 4 °C. Then, samples were dehydrated in an ascending series of ethanol dilutions in water as follows: 30% (4 h), 50% (4 h), 70% (overnight), 90% (2 h), and 100% (1 h). Once dehydrated, samples were dried in a Leica EM CPD300 automated critical point dryer, coated with platinum for 30 s in a Leica EM MED020 sputter coater, and mounted and observed in a ZEISS Ultra-55 scanning electron microscope operating at 2.0 kV.

### 4.4. Confocal Microscopy and FRET

Callose staining and *cameleon* and *WUS*-reporter lines were observed in a Zeiss 780 Axio Observer (Zeiss, Oberkochen, Germany) confocal laser scanning microscope. First, IZEs were cultured in solid E5 medium for five days. For callose staining, samples were then stained with 0.1% aniline blue in phosphate buffer for 1 h [15] and observed exciting at 405 nm and recording the 422–577 nm emission. For observation of *cameleon* and *WUS*-reporter lines, cultured IZEs were transferred to a microscope slide and mounted in 50 µL of 1.5% liquid low melting point agarose (SeaPlaque, Duchefa, Haarlem, Netherlands). Samples were immediately covered with a cover glass, solidified at room temperature, and observed. For dsRED visualization of *WUS*-reporter lines, samples were excited at 561 nm and the 563–618 nm emission was recorded. For FRET visualization of *cameleon* lines, samples were excited at 405 nm and emission was recorded between 490–570 nm. CFP was excited at 405 nm and emission was recorded between 440–488 nm. YFP was excited at 514 nm and emission was recorded between 518–570 nm. Image treatment and calculation of fluorescence emission ratios were performed as previously described [45]. For imaging, the FRET (YFP/CFP) ratio was defined as the ratio between YFP and CFP emissions (480/535 nm).

For all cases, Ca^2+^ levels were defined as very low, low, moderate, high, or very high according to the colorimetric scale based on the FRET ratio images. Very low Ca^2+^ levels corresponded to dark blue colors, low levels to light blue, moderate to green–yellow, high to orange–red, and very high levels to white color. Image analysis was performed using the FIJI software v 1.53t [46].

### 4.5. Ca^2+^ Modulators and Callose Inhibitor

To modulate the intracellular Ca^2+^ levels, we used CaCl_2_, the Ca^2+^ ionophore A23187, inositol 1,4,5-trisphosphate (InsP_3_), ethylene glycol-bis(β-aminoethyl ether)-N,N,N′,N′-tetraacetic acid (EGTA), chlorpromazine, and N-(6-Aminohexyl)-5-chloro-1-naphthalenesulfonamide hydrochloride (W-7). To inhibit callose deposition, 2-deoxy-D-glucose was used. Stocks of CaCl_2,_ InsP_3_, EGTA, chlorpromazine, and 2-deoxy-D-glucose were prepared in sterile water. Stocks of ionophore A23187 and W-7 were prepared in DMSO following manufacturer instructions. Different concentrations of each compound were applied to IZE in vitro cultures, as described in Results. Control plates were prepared with the corresponding solvent concentration. Fifteen days after initiation, the percentage of embryogenic IZEs out of the total was calculated by field counting IZEs in microscopic images of culture dishes [47].

### 4.6. Statistical Analysis

Statistical analysis was performed using StatGraphics. The results of Ca^2+^ chemical modulation were analyzed performing an ANOVA and LSD test (*p* ≤ 0.05). For non-homocedastic samples, data were transformed with the arcsine transformation or the square root of the arcsine transformation to stabilize variance.

## Figures and Tables

**Table 1:** Composition of the in vitro culture media used for induction of somatic embryogenesis. GB5: Gamborg basal medium + B5 vitamins [42]. MS: Murashige and Skoog basal medium + MS vitamins [43]. For all cases, pH was adjusted to 5.8 and media were autoclaved for 20 min at 121 °C. All basal media and other medium components were purchased from Duchefa (Netherlands).

	Induction	Germination
GB5 (g/L)	3.16	
MS (g/L)		4.6
Sucrose (%)	2	2
2,4-D (mg/L)	1.1	
Plant agar (%)	0.8	0.8

## Data Availability

All data supporting the findings of this study are available within the paper.

## Supplementary Materials

The following supporting information can be downloaded at: https://www.mdpi.com/article/10.3390/plants12051021/s1, Supplementary Figure S1. Comparison of the embryogenic response of wild type (Col-0) and *cameleon* lines; Supplementary Figure S2. FRET imaging of Ca^2+^ signaling during the induction of somatic embryogenesis in YC3.6-Bar *cameleon Arabidopsis* IZEs.
