# Supplementary material for: Calcium Dynamics, WUSCHEL Expression and Callose Deposition during Somatic Embryogenesis in Arabidopsis thaliana Immature Zygotic Embryos
Source: Plants (Basel). 2023 Feb 23;12(5):1021. doi: 10.3390/plants12051021 (PMC10005541; doi:10.3390/plants12051021)
Supplement: Supplementary file 1 [file plants-12-01021-s001.zip › plants-2196956-supplementary.pdf]

Supplementary figures

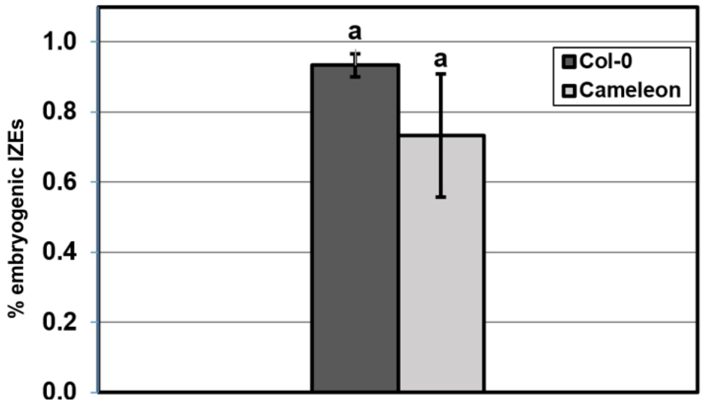

Figure S1. Comparison of the embryogenic response of wild type (Col-0) and *cameleon* lines, expressed as the percentage of embryogenic IZEs produced (% embryogenic IZEs) out of the total of embryos cultured. Same letters represent absence of significant differences according to the LSD test.

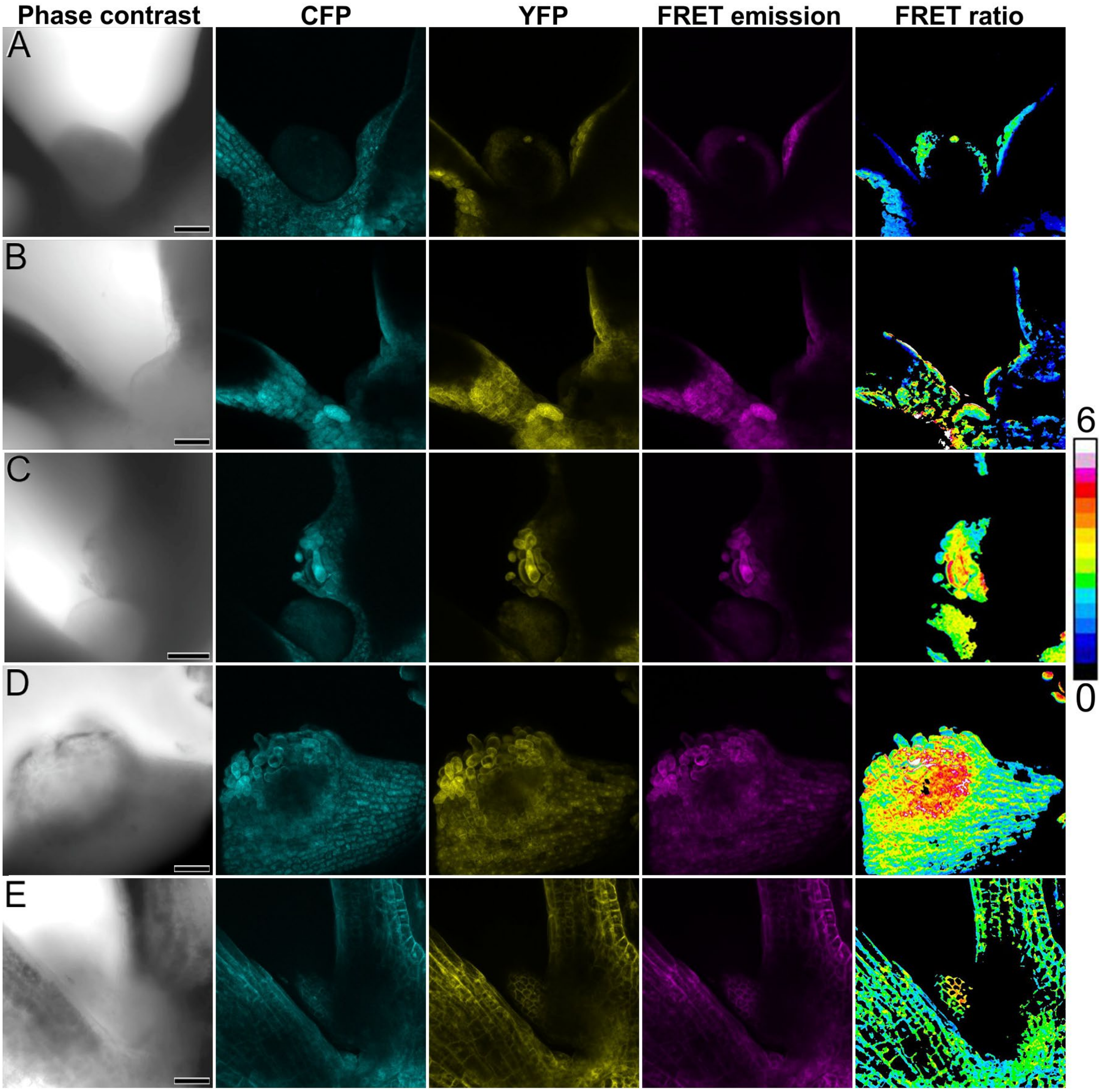

Figure S2. FRET imaging of Ca^{+2} signaling during the induction of somatic embryogenesis in YC3.6-Bar *cameleon* arabidopsis IZEs. Each set of images show the same stages shown in Fig. 4 imaged by phase contrast, CFP, YFP, FRET emission fluorescence and FRET (YFP/CFP emissions) ratio. The LUT bar displays the false coloration of FRET ratios. A: Shoot apical meristem and proximal region of the cotyledons, showing increased Ca^{+2} levels in the outermost cell layer of the shoot apical meristem and in the epidermis of the adaxial proximal cotyledon region. B: Cells of the mesophyll region of the cotyledon. C: Shoot apical meristem and a protrusion at the adaxial proximal cotyledon region. D: Large protrusion at the adaxial proximal region of the cotyledon with a radial gradient of Ca^{+2} levels. E: Shoot apical meristem and proximal region of the cotyledons of an IZE cultured in non-embryogenic conditions showing a homogeneous Ca^{+2} distribution. Bars: 60 μm.
